# Tertiary Syphilis-Induced Ocular Syphilis Complicated by Retinal Detachment

**DOI:** 10.7759/cureus.81240

**Published:** 2025-03-26

**Authors:** Arslan Khan, Stephen DiGiuseppe, Moneeb Mustafa

**Affiliations:** 1 Osteopathic Medicine, Edward Via College of Osteopathic Medicine, Monroe, USA; 2 Microbiology, Edward Via College of Osteopathic Medicine, Monroe, USA; 3 Internal Medicine, Rapid Regional Medical Center, Alexandria Louisiana, USA

**Keywords:** ocular syphilis, retinal detachment, syphilis, tertiary syphilis, vision loss

## Abstract

Tertiary syphilis can cause eye diseases such as ocular syphilis and retinal detachment. Herein, we present a case of a 28-year-old male with no reported past medical history. He presented to the emergency department with complaints of pain, blurriness, and vision loss in his left eye, leading to a fall during a recent ophthalmologist visit. Diagnosis typically relies on detailed clinical examination, lumbar punctures, and serological testing to diagnose and manage the patient's condition. The results of these tests led to the diagnosis of tertiary syphilis-induced ocular syphilis complicated by retinal detachment, which was treated with penicillin G intravenously (IV) daily. The patient was transferred to a long-term acute care facility with an ophthalmologist follow-up post-admission. This case highlights the importance of recognizing and emphasizing timely diagnosis of ocular syphilis and proper disease management as a crucial factor in preventing permanent vision loss.

## Introduction

Tertiary syphilis is the late stage of infection with *Treponema pallidum* (*T. pallidum*), a gram-negative spirochete bacterium, and is characterized by the development of destructive lesions known as gummas in various organs and tissues [1]. Ocular involvement, termed ocular syphilis, is a rare but serious manifestation of secondary or tertiary neurosyphilis. Syphilis progresses through distinct clinical stages, each characterized by unique pathophysiological mechanisms.

In primary syphilis, the initial response to *T. pallidum* invasion is the development of a solitary, nontender chancre, most commonly in the genital region. However, extragenital lesions can also occur on the digits, nipples, tonsils, and oral mucosa. These lesions arise at sites of direct inoculation and may be associated with tender or nontender lymphadenopathy. The incubation period ranges from 10 to 90 days, with a median of 21 to 25 days before lesion formation [1]. The chancre results from localized endothelial damage and the subsequent inflammatory response [1]. Even in the absence of treatment, these lesions resolve spontaneously without scarring.

If left untreated, primary syphilis progresses to secondary syphilis due to hematogenous dissemination of *T. pallidum*. This stage presents with a wide range of clinical manifestations, including mucocutaneous involvement, such as a macular rash, condyloma lata, and lesions on the hands and feet. Systemic symptoms such as headache, myalgia, arthralgia, pharyngitis, hepatosplenomegaly, and malaise are also common. The immune-mediated response in this stage often leads to diffuse lymphadenopathy and alopecia [1]. Despite its widespread presentation, secondary syphilis lesions also resolve spontaneously if left untreated, leading to the latent phase.

The latent phase of syphilis is clinically asymptomatic, with the infection only detectable through serologic testing. It is divided into early latent syphilis, referring to infection acquired within the past year, and late latent syphilis, which persists for over a year [1]. While many individuals remain asymptomatic in this phase, some will progress to tertiary syphilis.

Tertiary syphilis occurs years to decades after initial infection and is marked by chronic inflammatory damage affecting multiple organ systems. Among these, the most prevalent tertiary syphilis are cardiovascular syphilis, gummatous syphilis, and central nervous system (CNS) syphilis, also referred to as neurosyphilis [1]. Cardiovascular involvement may lead to aortitis and aortic aneurysm, while late neurosyphilis can manifest as tabes dorsalis or general paresis. Late benign syphilis, also known as gummatous syphilis, is characterized by granulomatous lesions that can affect various tissues. The prolonged immune response and tissue destruction in this stage often result in irreversible organ damage.

It is important to note that neurosyphilis can occur at any stage of the infection (early or late), although the presentation can be different at each stage. Syphilitic uveitis is the most common ocular manifestation of syphilis, and although rare, it can result in irreversible vision loss [2]. According to a study on the national incidence of syphilitic uveitis-related hospital admissions, the admission rate in the United States was reported as 0.15 per 100,000 population [2]. The incidence was lowest in 2011 (0.08 per 100 000 population) and showed an increasing trend over the years, with the highest incidence in 2019 (0.23 per 100 000 population) [2]. Risk factors are categorized into two distinct populations: men who have sex with men (MSM) and women [3,4]. Risk factors for MSM who contract syphilis include methamphetamine use, acquiring sexual partners from social media, and patients positive for human immunodeficiency virus (HIV) [3,5]. Risk factors related to women acquiring syphilis include recent injection drug use, including heroin, methamphetamines, or crack/cocaine, which leads to higher rates of syphilis [5,6]. Management depends on the patient’s HIV status, if the disease is in the latent symptomatic or asymptomatic stage, and if the patient has any penicillin allergies. Specific algorithms have been outlined for the workup and treatment of neurosyphilis based on the management criteria mentioned earlier [7].

## Case presentation

A 28-year-old male with no significant past medical history was transferred to the emergency department (ED) from an ophthalmologist clinic with concerns about late-stage tertiary syphilis. He presented with sudden onset of pain, blurry vision, multiple floaters, and vision loss in his left eye. While at the clinic, he had a fall due to his visual disturbances. The ophthalmologist’s findings, suggestive of retinal detachment, raised clinical suspicion for ocular syphilis, prompting referral to the ED. Upon arrival, the patient reported a painless genital lesion two years ago that resolved spontaneously. He denied systemic symptoms such as fever, weight loss, or rashes but admitted to multiple sexual partners. The physical exam revealed an alert, mildly distressed patient with a dilated left pupil and reduced visual acuity of 20/200, while the right eye showed normal acuity at 20/20. The dilated fundoscopic exam revealed extensive retinal detachment in the left eye, with subretinal fluid and undulating folds, consistent with macular involvement. The fluorescein angiography images illustrate hallmark features of inflammatory etiology, including diffuse retinal vascular leakage, hyperfluorescence of the optic disc, and focal retinal inflammation. These findings reflect the widespread and severe inflammatory process caused by tertiary syphilis, emphasizing the critical need for timely diagnosis and treatment.

**Figure 1:**
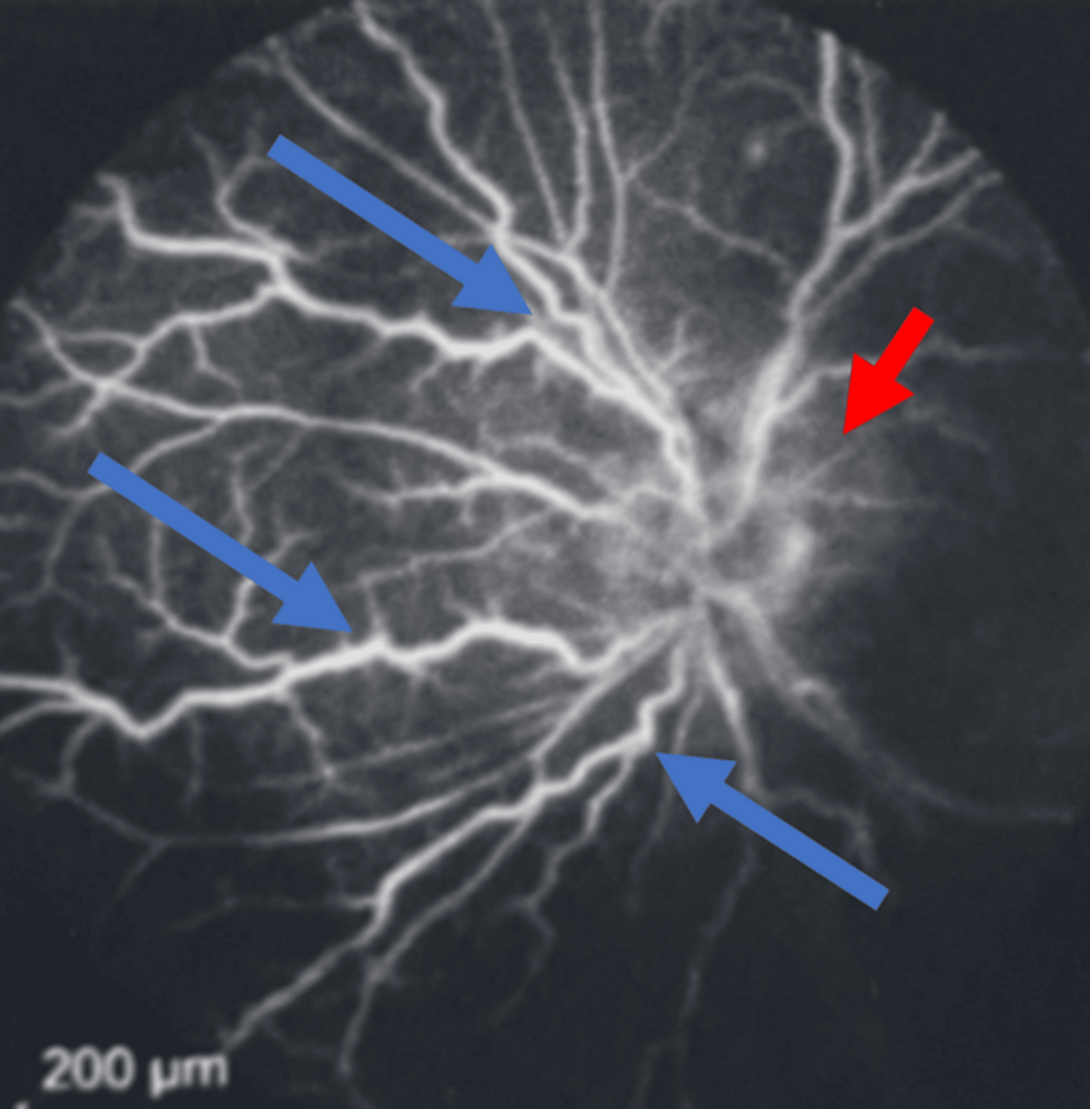
Fluorescein angiographic imaging of the patient's right eye Fluorescein angiographic imaging of the patient's right eye was used to reveal diffuse hyperfluorescence of the retinal vasculature denoted by the blue arrows, indicative of vasculature inflammation due to an infectious etiology. The red arrow denotes the significance of the optic disc appearing unaffected, with no evidence of leakage or hyperfluorescence at the level of the disc.

The imaging correlated with the patient’s symptoms of blurred vision and photopsia, confirming the posterior uveitis and vasculitis as the primary contributors to visual disturbances. Fluorescein angiographic imaging of the patient’s right eye was used to reveal diffuse hyperfluorescence of the retinal vasculature, denoted by the blue arrows, indicative of vasculature inflammation due to an infectious etiology (Figure 1). The red arrow points out the significance of the optic disc appearing unaffected, with no evidence of leakage or hyperfluorescence at the level of the disc (Figure 1).

**Figure 2:**
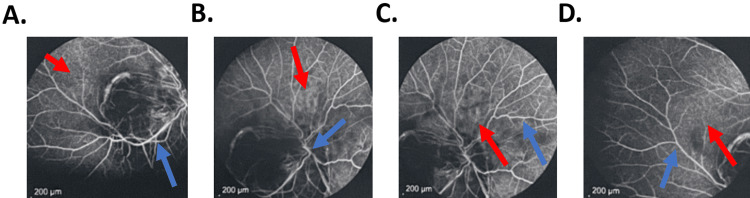
Fluorescein angiographic imaging of the left eye (affected eye) Fluorescein angiographic imaging of the left eye (affected eye) was used as the diagnostic study of choice to characterize the symptomatic findings. (A) The red arrow indicates hyperfluorescence due to retinal vascular leakage, which is characteristic of active inflammation in panuveitis. (B) The red arrow highlights areas of optic disc hyperfluorescence, indicating optic disc leakage secondary to inflammation. This finding reflects possible optic neuritis or perineural involvement. (C) The red arrow points to a localized area of hyperfluorescence in the mid-retina. (D) The red arrow mark diffuse staining and leakage. (A–D) The blue arrows exemplified in each of these images denoting diffuse hyperfluorescence of the retinal vasculature, indicative of vasculature inflammation.

Fluorescein angiographic imaging of the left eye (affected eye) was used as the diagnostic study of choice to characterize the symptomatic findings (Figures 2A-2D). The red arrow in Figure 2A indicates hyperfluorescence due to retinal vascular leakage, which is characteristic of active inflammation in panuveitis. The red arrow in Figure 2B highlights areas of optic disc hyperfluorescence, indicating optic disc leakage secondary to inflammation. The red arrow in Figure 2C points to a localized area of hyperfluorescence in the mid-retina, suggestive of focal inflammatory activity. This is indicative of vascular leakage and potential disruption of the blood-retinal barrier due to active inflammatory disease. Lastly, the red arrow in Figure 2D marks diffuse staining and leakage consistent with the widespread retinal vascular involvement typically observed in severe cases of panuveitis. The blue arrows denote diffuse hyperfluorescence of the retinal vasculature, indicative of vasculature inflammation (Figures 2A-2D).

Cardiovascular, respiratory, and neurological exams were unremarkable, and the abdominal exam showed no organomegaly. Sensory and motor function in both upper and lower extremities were intact, with no gait abnormalities or reflex issues observed. Given the patient’s ocular findings and sexual history, there was a strong suspicion of ocular syphilis secondary to tertiary syphilis.

The patient was promptly started on penicillin G, 4 million units intravenously (IV) every four hours, and sodium chloride 0.9% IV for hydration. Ophthalmology and Infectious Disease (ID) consultations were requested, and the patient was admitted for inpatient care. A full workup was initiated, including complete blood count (CBC), comprehensive metabolic panel (CMP), blood cultures, HIV and hepatitis screening, rapid plasma reagin (RPR) titers, and a lumbar puncture to assess for neurosyphilis (Table 1). By day 2 of hospitalization, the patient reported some improvement in his eye pain, though his visual symptoms persisted. Lumbar puncture results were pending, but daily labs, including CBC and CMP, remained stable. Infectious Disease followed closely, and the decision was made to continue IV penicillin for 14 days. On day 5, rapid plasma reagin (RPR) titers returned positive at 1:16, confirming syphilis infection. HIV, hepatitis panel, lumbar puncture results, and methicillin-resistant *Staphylococcus aureus* (MRSA) cultures were all negative.

**Table 1:** Summary of relevant investigations Ig: Immunoglobulin.

Parameters	Values	Reference Range
Complete blood count (CBC)	8500 cells/μL	4000 - 11000 cells/μL
Alanine aminotransferase (ALT)	46 U/L	4 - 36 U/L
Aspartate aminotransferase (AST)	19 U/L	0 - 35 U/L
Treponemal pallidum antibody	Reactive	Non-Reactive
Rapid plasma reagin (RPR)	Reactive	Non-Reactive
Rapid plasma reagin (RPR) titer on day 5	1:16	< 1:1
Hepatitis A IgM antibody	Negative	Negative
Hepatitis B surface antigen	Negative	Negative
Hepatitis B Core IgM antibody	Negative	Negative
Hepatitis C antibody	Negative	Negative
Hepatitis interpretation	Negative	Negative
HIV 1 & 2 antibody & HIV 1 antigen	Negative	Negative

Given the confirmed diagnosis of tertiary syphilis with ocular involvement, the treatment plan remained unchanged. A social worker was consulted to arrange the patient’s discharge to a long-term acute care facility to complete his IV antibiotic regimen. Infectious Disease recommended administering benzathine penicillin G intramuscularly following completion of the IV course. A follow-up ophthalmology appointment was scheduled for one week post-discharge to monitor the patient’s retinal detachment and ongoing visual symptoms.

## Discussion

Tertiary syphilis, notably ocular syphilis, represents a relatively uncommon occurrence in clinical practice in the post-antibiotic era. However, the increasing prevalence reported of the disease among people with HIV in the last several years may become more common [8-10]. One report suggested that ocular syphilis may be linked to specific strains of *T. pallidum* [11]. The difficulty arising with recognizing and treating syphilis is the bacteria's ability to evade the host immune response and replicate to establish the primary infection in the localized tissue. As *T. pallidum* forms the ulcerative lesion, some bacteria also spread to initiate infection in nearby lymph nodes [1,12]. This may create the falsified perception that the infection has resolved on its own because of the host's innate and adaptive immune response to the localized primary lesion. While this initially results in temporary relief, the spirochetes persist and continue to replicate until they disseminate throughout the body, potentially affecting any organ system and resulting in diverse clinical manifestations, including ocular involvement [1,12].

Given that patients with retinal detachment often present with no systemic symptoms suggestive of syphilis, a considerable number of patients are only diagnosed clinically [13]. Most patients with symptoms of ocular syphilis are often also HIV positive. However, confirming this infectious cause remains challenging due to its rarity and limited awareness in patients without any history of HIV infection or other symptomatic manifestations of syphilis. Patient history, a high clinical suspicion, and serological testing remain the mainstay of clinical diagnosis for ocular syphilis. Patients with ocular syphilis can develop anterior uveitis, posterior uveitis, or panuveitis, which is often granulomatous [10,14]. Most patients with ocular syphilis also develop diminished visual acuity secondary to posterior uveitis [10,14]. Posterior uveitis can then proceed to retinal necrosis and detachment.

Panuveitis with retinal detachment, along with potential evidence of optic neuritis at the optic disc as observed on fluorescein angiography, represents some of the key ocular findings commonly associated with late secondary and tertiary syphilis [13]. What sets our case apart from others is the absence of systemic involvement, as well as the lack of the maculopapular or pustular rash typically characteristic of secondary syphilis. This raises the question of whether the lesion may have originated at the level of the eyelid or an iris nodule that went undiagnosed due to a lack of healthcare follow-up. The scientific literature lacks an explanation directly addressing the reasons for a shortened incubation period in patients with ocular syphilis as related to our case. The progression to tertiary stages is generally due to untreated or inadequately treated primary and secondary syphilis. Ocular involvement can occur at any stage of syphilis, but when it presents in the context of tertiary syphilis, it indicates a prolonged course of untreated infection. However, the absence of an iris nodule does not rule out secondary syphilis as a potential cause, given its occurrence in other reported cases [13].

An additional notable finding in this case, which differs from many other instances of ocular syphilis, is the unilateral involvement of the left optic nerve (as shown in Figure 2B), suggesting potential optic neuritis or perineural involvement associated with the later stages of disease progression. The retinal vasculature in the right eye also exhibits inflammatory changes like those observed in the left eye on fluorescein angiography (indicated by the red arrows in Figure 1), while the unaffected optic disc suggests signs of early infection. This raises suspicion for slow disease progression as it migrates from a unilateral presentation to a more diffuse bilateral encroachment if left untreated. Figure 2C highlights the localized area of hyperfluorescence in the mid-retina, suggestive of focal inflammatory activity. This is indicative of vascular leakage and potential disruption of the blood-retinal barrier due to active inflammatory disease. Figure 2D shows diffuse staining and leakage consistent with the widespread retinal vascular involvement typically observed in severe cases of panuveitis. These images focus our attention on the later stages of disease progression, with the blue arrows exemplified in each of these images denoting diffuse hyperfluorescence of the retinal vasculature, indicative of vasculature inflammation due to an infectious etiology (Figures 2A-2D). 

Guidelines for the management of ocular syphilis follow the same steps as neurosyphilis as recommended by the Centers for Disease Control (CDC). As recommendations evolve continuously, a recent update from the CDC states that lumbar puncture is no longer necessary in patients with ocular syphilis in the absence of neurological symptoms or signs, although it might assist in making the diagnosis [13]. The Cerebro Spinal Fluid-Venereal Disease Research Laboratory test (CSF-VDRL) has been considered the gold standard for diagnosing neurosyphilis [15]. Although it has proven to have high specificity, its low sensitivity excludes it from being an adequate screening test for neurosyphilis [15]. The complicated process associated with performing the test itself adds limitations to interpreting the results. Furthermore, other diseases, central nervous system malignancies, and CSF contamination by blood from events such as craniocerebral trauma may lead to a false-positive CSF-VDRL test result in patients with syphilis, contributing to low sensitivity [15]. The recommended regimen is IV penicillin G at a dose of 3-4 million units every four hours (or as a continuous infusion of 18-24 million units per day) for 10 to 14 days [16]. If the patient has a penicillin allergy, they should first be assessed to see if they can be desensitized due to their high effectiveness in managing the disease. The recommended alternative regimen for a patient who cannot tolerate desensitization of penicillin is ceftriaxone 2 g IV daily for 10 to 14 days if the patient is able to tolerate cephalosporin as provided by CDC guidelines [17].

## Conclusions

Herein, we present a case of ocular syphilis, a known complication of tertiary syphilis that needs emergent attention due to its preventative nature in avoiding permanent vision loss. The diagnosis of ocular syphilis often poses a formidable challenge owing to its rare occurrences and the presence of nonspecific clinical findings in patients with a negative HIV status. In such instances, it becomes crucial to promptly employ serological testing, such as RPR titers, along with a thorough patient history aligned with the presenting symptoms. In certain scenarios, lumbar punctures may also be necessary to definitively confirm the diagnosis. Management strategies for patients with ocular syphilis primarily revolve around antibiotic treatment achieved using high-dose intravenous or intramuscular penicillin. Therefore, a comprehensive strategy that integrates vigilant diagnostic evaluation and customized symptomatic treatment is essential for effectively addressing this uncommon progression of disease.
